# Specimens from Biopsies of Colorectal Polyps Often Harbor Additional Diagnoses

**DOI:** 10.1155/2013/570526

**Published:** 2013-12-24

**Authors:** Shefali Chopra, Mark Li-cheng Wu

**Affiliations:** Department of Pathology and Laboratory Medicine, University of California Irvine School of Medicine, Irvine, CA 92868, USA

## Abstract

*Objectives*. The utility of examining specimens from colorectal biopsies of polyps for nonneoplastic diseases is currently unknown. Our objectives were to characterize such additional diagnoses that could be rendered. *Methods*. We retrospectively and prospectively reviewed specimens from endoscopic biopsies of colorectal polyps obtained during routine screening or surveillance. *Results*. 17 of 168 specimens (10.1%) contained additional diagnoses, including schistosomiasis, eosinophilic colitis, intestinal spirochetosis, melanosis coli, and other entities. These findings were easily overlooked because they often affected mucosa that was spared by the polyps or were often evident only at high magnification. Schistosomiasis, eosinophilic colitis, and intestinal spirochetosis were clinically occult. *Conclusions*. Specimens from biopsies of colorectal polyps often harbor other diagnoses, in addition to polyps, and can be simultaneously screened for polyps and examined for nonneoplastic diseases. Detection of other diagnoses in addition to polyps requires awareness, examination at high magnification, and examination of areas spared by the polyps.

## 1. Introduction

Specimens from endoscopic biopsies of putative colorectal lesions usually show traditional nonserrated adenomas, serrated polyps, or variants of normal mucosa. These entities generally are easily and rapidly diagnosed at low magnification. Consequently, pathologists are tempted to examine these specimens quickly and only at low magnification, to assume that these specimens will harbor only polyps or variants of normal mucosa, and to refrain from examining these specimens further at high magnification once polyps are diagnosed. Directed review of these specimens might reveal other diagnoses, in addition to polyps. Pathologists might overlook such second diagnoses for various reasons. However, such second diagnoses might be significant. We reviewed specimens from biopsies of colorectal polyps to characterize second diagnoses that could be rendered in this setting and to demonstrate that these specimens could be examined for nonneoplastic diseases.

## 2. Materials and Methods

The study was approved by the Institutional Review Board of the University of California, Irvine, USA, on November 7, 2005 as protocol HS number: 2005-4646. The study was carried out in 2 phases. The first phase was designed to determine prevalence, and the second phase was designed to determine incidence.

During the first phase, consecutive specimens from endoscopic colorectal biopsies of polyps accessioned at our institution during a 1-month interval, from July 1, 2003 to August 1, 2003, were retrospectively reviewed by both of us, who are gastrointestinal pathologists, with special effort to render second diagnoses in addition to polyps. Only specimens that had polyps, broadly defined as protruding pathologic mucosal lesions [1], confirmed histologically were eligible for review. All original diagnoses were made by 5 other pathologists, who were general surgical pathologists. These pathologists were unaware of our study. During the second phase, specimens from 100 consecutive histologically confirmed polyps obtained from endoscopic colorectal biopsies accessioned to 1 of us (M. L.-c. Wu) at our institution during the course of normal signout were prospectively accrued during the interval July 1, 2007–September 11, 2007. These specimens were examined with knowledge of our study and with special effort to render second diagnoses in addition to polyps. For both phases, this special effort consisted of examining all sections on each slide, at low magnification and high magnification, and examining the entire surface area of each section including mucosa affected by polyps and mucosa spared by polyps. Specimens from both phases with additional diagnoses were reviewed by both of us for confirmation.

Pathology reports and requisitions for all specimens were reviewed. Corresponding reports from endoscopy were reviewed for each specimen, in either phase, for which additional diagnoses were rendered. Diagnoses were considered clinically occult if the requisitions or corresponding reports from endoscopy lacked the mention of the diagnoses or lacked the mention of findings that could be reasonably attributed to the diagnoses. Excluded from the entire study were specimens from biopsies of putative lesions that lacked polyps histologically. All specimens were processed routinely, formalin-fixed, and paraffin-embedded. All histologic sections were cut 4 microns thick and stained with hematoxylin and eosin. Microscopy was performed with a conventional multiheaded optical microscope (BX45, Olympus, Melville, NY, USA). All diagnoses were rendered according to standard criteria [1–4].

## 3. Results

For the first phase, 78 specimens were diagnosed as polyps by original pathologists in the 1-month interval. We reclassified 3 hyperplastic polyps and 1 tubular adenoma as normal mucosa and excluded these specimens from further study. We reclassified 1 mucosal prolapse, 1 hyperplastic polyp, and 1 inflammatory polyp, as leiomyoma, sessile serrated adenoma, and tubular adenoma, respectively. We confirmed all other original diagnoses. A few specimens had multiple polyps. The first phase therefore eventually consisted of 74 specimens, with 78 histologically confirmed polyps. These polyps were from 52 patients, ages 43 to 74, including 31 men and 21 women. The 78 polyps included 48 polyps in men and 30 polyps in women.

**Figure 1:**
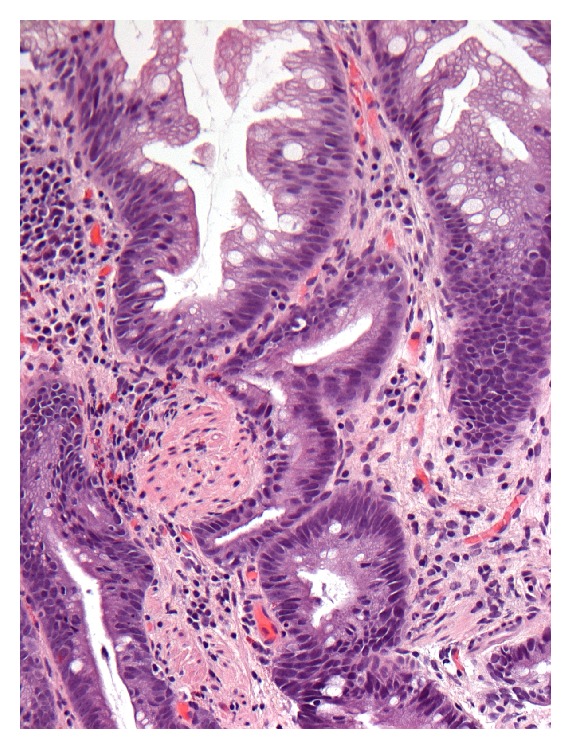
Mucosal prolapse in specimen with hyperplastic polyp. Only small focus of smooth muscle present (left of center) (H&E, ×200).

**Figure 2:**
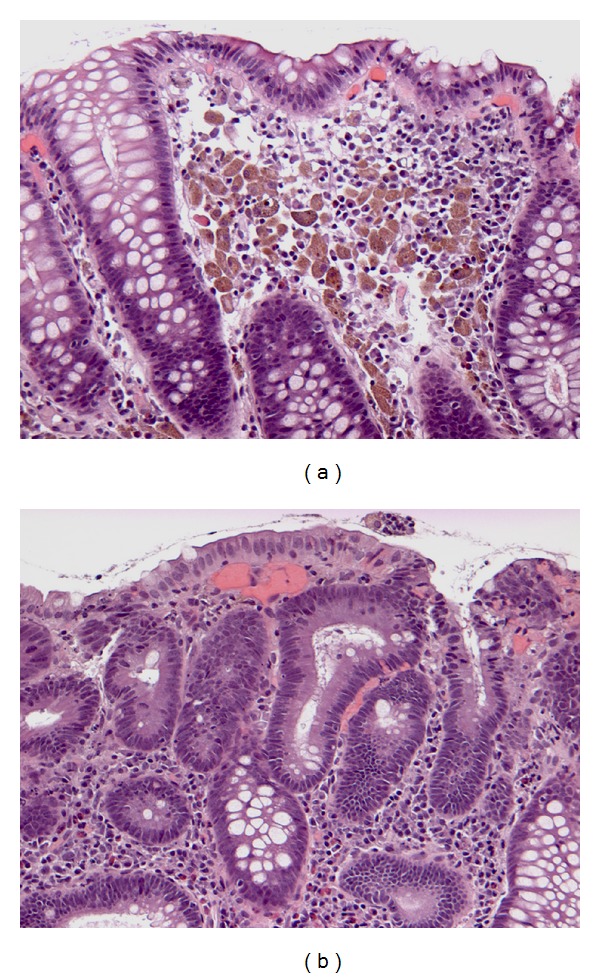
Melanosis coli in specimen with tubular adenoma. (a) Melanosis affects nonpolypoid mucosa (H&E, ×200) and (b) spares adenoma (H&E, ×200).

**Figure 3:**
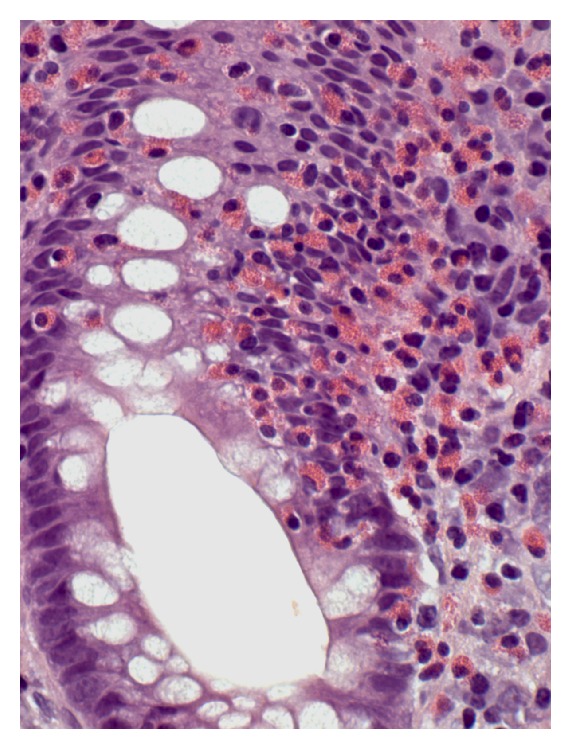
Eosinophilic colitis in specimen with tubular adenoma. Nonpolypoid mucosa contains abundant eosinophils in lamina propria, eosinophilic cryptitis, and absence of neutrophils (H&E, ×600).

Of the 74 specimens, additional diagnoses were retrospectively detected by us in 7 specimens (9.5%). These diagnoses included the following: mucosal prolapse (Figure 1) (in 2 specimens with hyperplastic polyps and 1 specimen with tubular adenoma), melanosis coli (Figures 2(a) and 2(b)) (in 2 specimens with tubular adenomas), hyperplastic polyp (in 1 specimen with tubular adenoma), and eosinophilic colitis (Figure 3) (in 1 specimen with tubular adenoma).

The patient for whom we diagnosed eosinophilic colitis was asymptomatic. With this caveat, we rendered the diagnosis of eosinophilic colitis based on compelling morphology that satisfied recently proposed criteria [3]. Microscopy showed the entire specimen, including areas remote from the polyp, to have an extremely high density of eosinophils with greater than 120 eosinophils per 600x field, frequent eosinophilic cryptitis and crypt abscesses, degranulated eosinophils, submucosal eosinophils, and absence of neutrophils in nonpolypoid mucosa.

Mention of all of these additional diagnoses, except for eosinophilic colitis, was absent from the original pathology reports. Therefore, 6 of 7 additional diagnoses were potentially overlooked by original pathologists.

For the second phase, the 100 polyps were accrued after examining 94 specimens. A few specimens had multiple polyps. The 94 specimens were from 55 patients, including 27 men and 28 women, ages 45 to 82. The 100 polyps included 57 polyps in men and 43 polyps in women.

**Figure 4:**
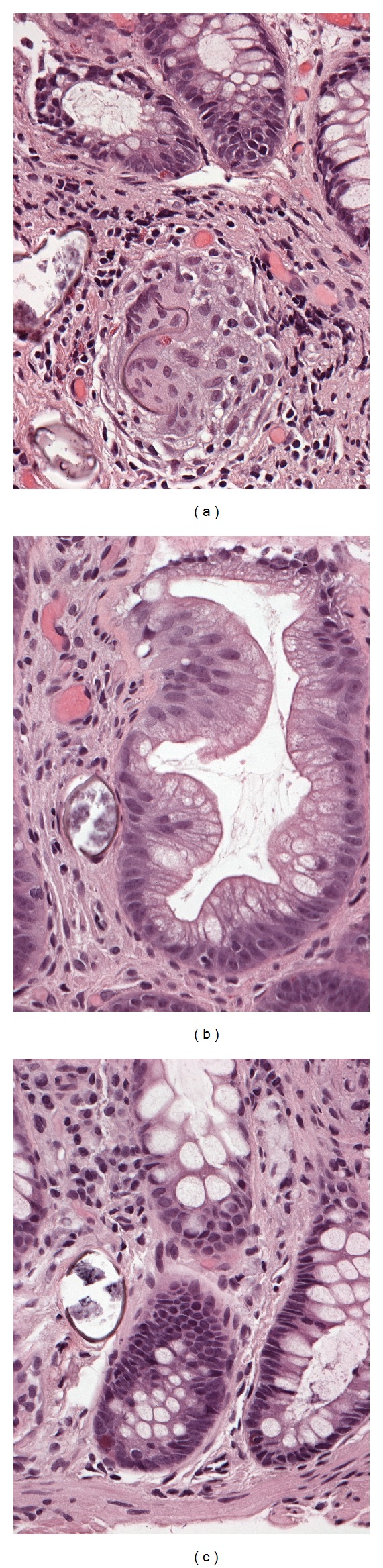
Schistosomiasis in specimens with adenocarcinoma and hyperplastic polyp. (a) Nonpolypoid mucosa beside adenocarcinoma (not shown) shows schistosomiasis and granuloma (H&E, ×400). (b) Schistosomiasis affects hyperplastic polyp (H&E, ×400) and (c) adjacent, otherwise normal nonpolypoid mucosa (H&E, ×400).

**Figure 5:**
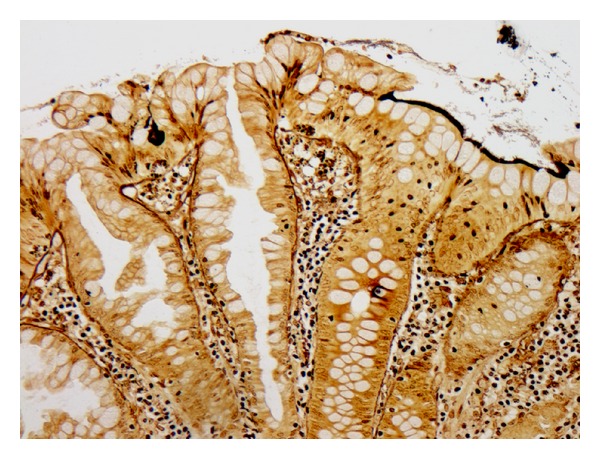
Intestinal spirochetosis in specimen with sessile serrated adenoma. Spirochetes colonize surface of otherwise normal mucosa present beside polyp (top right) and present focally between serrated crypts (top left) (Warthin-Starry, ×200).

Of the 94 specimens, additional diagnoses were prospectively detected in 10 specimens (10.6%) by 1 of us (M. L.-c. Wu). These diagnoses included the following: melanosis coli (in 4 specimens with tubular adenomas and 1 specimen with hyperplastic polyp), mucosal prolapse (in 2 specimens with hyperplastic polyps), schistosomiasis (Figures 4(a), 4(b), and 4(c)) (in 1 specimen with adenocarcinoma and 1 specimen with hyperplastic polyp), and intestinal spirochetosis (Figure 5) (in 1 specimen with sessile serrated adenoma), and they were confirmed retrospectively by both of us.

For the entire study, a final total of 168 specimens were included, and 17 additional diagnoses were rendered, for a rate of 10.1%. Clinical information obtained from pathology requisitions and corresponding notes from endoscopy indicated that all endoscopies were performed for screening or surveillance for polyps and that all patients were asymptomatic.

Several factors potentially contributed to difficulty in rendering additional diagnoses. The additional diagnostic findings were easily overlooked in many scenarios (Figures 1, 2(a), 2(b), 3, 4(a), 4(b), 4(c), and 5), including when the findings were small, focal, in nonpolypoid mucosa, in otherwise normal mucosa, or clinically occult.

Tables 1, 2, and 3 summarize the histology and distribution of the polyps and the additional diagnoses.

## 4. Discussion

Specimens taken for the purpose of evaluating 1 disease may contain additional, incidental diagnostic findings, which may be related or unrelated to the primary disease. These additional diagnoses are easily overlooked, because of many reasons: (1) additional diagnoses may be associated with morphologic changes that are considerably smaller than those of the primary disease and might only be appreciated at high magnification or by purposefully examining the slide for incidental findings; (2) attention of pathologists is usually directed toward the primary disease due to habit and due to bias from the clinical request, and hence attention is diverted away from incidental findings; (3) incidental findings may occupy seemingly extraneous tissue, from the same organ or unrelated organs, that usually is histologically normal [5]; (4) even if incidental findings are detected, pathologists that lack expertise in a particular specialty may fail to recognize incidental findings as significant. It is important for pathologists to report such additional diagnoses because these additional diagnoses may have pathologic, endoscopic, or clinical significance or may be clinically occult. This process transforms these specimens into invaluable, cost-effective tools to detect unexpected diagnoses. Examining for additional diagnoses detects disease early, guides therapy, and eliminates morbidity and financial burden associated with repeat biopsies and advanced disease. Colonoscopy carries a 0.07% incidence of iatrogenic perforation [6] and costs approximately $3081 by itself but costs significantly more with biopsy, anesthesia, or immunohistochemistry or if performed on an inpatient [7].

The concept of purposefully examining specimens to detect other, secondary diagnoses has been studied in the setting of genitourinary pathology and hepatic pathology. Specimens from nephrectomies performed to stage primary renal neoplasia were recently shown to occasionally contain incidental nonneoplastic disease [8]. Specimens from prostatic core biopsies often contain incidental pieces of rectal mucosa that occasionally harbors clinically significant rectal pathology or causes diagnostic difficulty [5]. Specimens from hepatic core biopsies obtained for the purpose of grading and staging chronic viral hepatitis often contain other diagnoses [9]. To our knowledge, our study is the first to apply this concept to colorectal biopsies.

Our results show that the rate of additional diagnoses in specimens from biopsies of colorectal polyps in asymptomatic patients at time of screening or surveillance is approximately 10%. This rate is predictably similar to that found in a recent study, which detected abnormal histology in 11% of specimens from colorectal biopsies of patients with normal endoscopy and without diarrhea [10]. Furthermore, this rate is predictably much less than that found in a previous study, which detected abnormal histology in 32.1% of patients with normal colonoscopy and with chronic diarrhea [11].

Although some additional diagnoses had minor clinical impact, all had pathologic or endoscopic significance. For reasons yet to be elucidated, melanosis coli generally avoids adenomas, as our study demonstrated. Adenomas spared by melanosis coli appear endoscopically as pale patches, which help endoscopists locate adenomas [12]. Our study also demonstrated that intestinal spirochetosis and schistosomiasis may be asymptomatic, involve normal or abnormal mucosa, and involve polyps [13–19]. To our knowledge, our study is the first to report schistosomiasis coincidentally affecting a hyperplastic polyp. Although our case of eosinophilic colitis was easily diagnosed, caution must be exercised to avoid overdiagnosing colitis, because polyps commonly attract eosinophils and lymphocytes [20–22]. Mucosal prolapse may mimic hyperplastic polyps, sessile serrated adenomas, traditional nonserrated adenomas, inflammatory bowel disease, and other entities [4, 23].

Specimens from colorectal biopsies of polyps can and should be simultaneously screened for polyps and examined for additional diagnoses. Awareness of the possibility of second diagnoses, examination of nonpolypoid areas, and examination at high magnification are necessary to avoid overlooking these diagnoses.

## Figures and Tables

**Table 1:** Histology of polyps.

Histology	Phase 1	Phase 2
Tubular adenomas	49	34
Tubulovillous adenomas	2	10
Adenocarcinomas	2	2
Hyperplastic polyps	22	46
Sessile serrated adenomas	2	7
Leiomyomas	1	0
Neuroendocrine tumors	0	1

**Table 2:** Distribution of polyps.

Site	Phase 1	Phase 2
Cecum	9	11
Ascending colon	15	11
Hepatic flexure	1	3
Transverse colon	1	11
Splenic flexure	2	1
Descending colon	9	9
Sigmoid colon	12	28
Rectum	15	24
Unspecified	14	2

**Table 3:** Additional diagnoses detected at colorectal biopsy for polyps.

Additional diagnoses	Factors potentially contributing to diagnostic difficulty
Mucosal prolapse	Only small focus affected

Melanosis coli	Nonpolypoid mucosa preferentially affected
	Polyps relatively or completely spared

Eosinophilic colitis	Confusion with reaction to adenoma
	Clinically occult

Schistosomiasis	Minute ova
	Affected nonpolypoid mucosa otherwise normal or focally normal
	Clinically occult

Intestinal spirochetosis	Minute bacteria
	Confined to nonpolypoid mucosa
	Affected nonpolypoid mucosa otherwise normal
	Clinically occult
